# Temporal Dynamics of Co-circulating Lineages of Porcine Reproductive and Respiratory Syndrome Virus

**DOI:** 10.3389/fmicb.2019.02486

**Published:** 2019-11-01

**Authors:** Igor Adolfo Dexheimer Paploski, Cesar Corzo, Albert Rovira, Michael P. Murtaugh, Juan Manuel Sanhueza, Carles Vilalta, Declan C. Schroeder, Kimberly VanderWaal

**Affiliations:** ^1^Department of Veterinary Population Medicine, University of Minnesota, Saint Paul, MN, United States; ^2^Department of Veterinary and Biomedical Sciences, University of Minnesota, Saint Paul, MN, United States; ^3^School of Biological Sciences, University of Reading, Reading, United Kingdom

**Keywords:** PRRSV, epidemiology, ecology, evolution, multi-strain dynamics, emergence, outbreak

## Abstract

Porcine Reproductive and Respiratory Syndrome Virus (PRRSV) is the most important endemic pathogen in the U.S. swine industry. Despite control efforts involving improved biosecurity and different vaccination protocols, the virus continues to circulate and evolve. One of the foremost challenges in its control is high levels of genetic and antigenic diversity. Here, we quantify the co-circulation, emergence and sequential turnover of multiple PRRSV lineages in a single swine-producing region in the United States over a span of 9 years (2009–2017). By classifying over 4,000 PRRSV sequences (open-reading frame 5) into phylogenetic lineages and sub-lineages, we document the ongoing diversification and temporal dynamics of the PRRSV population, including the rapid emergence of a novel sub-lineage that appeared to be absent globally pre-2008. In addition, lineage 9 was the most prevalent lineage from 2009 to 2010, but its occurrence fell to 0.5% of all sequences identified per year after 2014, coinciding with the emergence or re-emergence of lineage 1 as the dominant lineage. The sequential dominance of different lineages, as well as three different sub-lineages within lineage 1, is consistent with the immune-mediated selection hypothesis for the sequential turnover in the dominant lineage. As host populations build immunity through natural infection or vaccination toward the most common variant, this dominant (sub-) lineage may be replaced by an emerging variant to which the population is more susceptible. An analysis of patterns of non- synonymous and synonymous mutations revealed evidence of positive selection on immunologically important regions of the genome, further supporting the potential that immune-mediated selection shapes the evolutionary and epidemiological dynamics for this virus. This has important implications for patterns of emergence and re-emergence of genetic variants of PRRSV that have negative impacts on the swine industry. Constant surveillance on PRRSV occurrence is crucial to a better understanding of the epidemiological and evolutionary dynamics of co-circulating viral lineages. Further studies utilizing whole genome sequencing and exploring the extent of cross-immunity between heterologous PRRS viruses could shed further light on PRRSV immunological response and aid in developing strategies that might be able to diminish disease impact.

## Introduction

Porcine reproductive and respiratory syndrome virus (PRRSV), the etiological agent of PRRS, is one of the most important endemic viruses affecting the swine industry in the United States (Holtkamp et al., 2013) and globally (Stadejek et al., 2013; VanderWaal and Deen, 2018). The economic impact of the disease in the United States has been estimated at $664 million annually (Holtkamp et al., 2013). Clinical signs in affected farms vary by viral variant and according to the farm’s production stage (e.g., breeding or growing herd), herd management, immune status, and other factors (Goldberg et al., 2000). Premature farrowing can occur in 5–30% of sows in an affected farm, and up to 35% of piglets are stillborn during an outbreak (Christianson and Joo, 1994). Piglets may be born with low weight and can present with lethargy and anorexia, which can lead to a mortality of more than 70% among piglets (Pejsak et al., 1997). PRRSV-infected pigs are also susceptible to secondary infections leading to poor average daily gain and feed conversion, further increasing production loss (Solano et al., 1997; Xu et al., 2010). Up to 40% of United States breeding herds experience outbreaks annually (Tousignant et al., 2015a) and control of the disease in the United States, Europe, and globally is challenging due to high levels of antigenic variability and its rapidly expanding genetic diversity (Frossard et al., 2013; Brar et al., 2015; Guo et al., 2018; Smith et al., 2018).

Porcine reproductive and respiratory syndrome virus was first recognized almost simultaneously in Europe (Wensvoort et al., 1991) and North America (Collins et al., 1992) in the late 1980s and early 1990s, but genetic differences suggested a much earlier evolutionary divergence between the North American and European viral types. Thus, PRRSV is divided into two major phylogenetic clades, PRRSV Type 1 (more prevalent in Europe) and Type 2 (more prevalent in North America) (Shi et al., 2010a, b; Stadejek et al., 2013). Within each clade, high levels of genetic and antigenic diversity exist and cross-protection is only partial (Roberts, 2003; Kim et al., 2013; Correas et al., 2017). Genetic similarities between PRRSV isolates have been used as a tool to understand disease transmission and epidemiology (Kapur et al., 1996; Wesley et al., 1998), and several different strategies have been used for classifying isolates of PRRSV into epidemiologically meaningful groups. For PRRSV Type 2, the most commonly used classification system is based on restriction fragment length polymorphisms (RFLP) and sequencing, both of which are typically based on the open reading frame 5 (*ORF5*) portion of its genome (Kapur et al., 1996; Wesley et al., 1998). The *ORF5* gene encodes for the major envelope protein (GP5), which plays a role in inducing virus neutralizing antibodies and cross-protection among PRRSV variants (Dea et al., 2000; Kim et al., 2013). RFLPs have been broadly adopted by the U.S. swine industry despite shortcomings, such as the fact that the genetic relationship between different RFLP types is unclear, the potential for two distantly related viruses to share the same RFLP type, and the instability of RFLP-typing when assessing isolates related to each other by as few as 10 animal passages (Cha et al., 2004). In 2010, a classification system based on the phylogenetic relatedness of the *ORF5* portion of the virus’s genome was proposed (Shi et al., 2010a, b). This classification system aggregates isolates into phylogenetic lineages based on the ancestral relationships and genetic distance among isolates. Using this system, nine different lineages were described within PRRSV Type 2, each of which was estimated to have diverged between 1980 and 1992 (Shi et al., 2010b). Phylogeny-based classification of organisms is seen as the most powerful and robust instrument for distinguishing between variants of a viral population (Hungnes et al., 2000) and has been used in the study of other viral diseases (Liu et al., 2009). Phylogeny-based classification of PRRSV, rather than RFLP profiling, is expected to provide fewer ambiguities and more insight into the evolutionary relatedness amongst different variants. While the existence of PRRSV lineages is well established, the dynamics of their co-circulation within a given region has not been well documented.

Vaccination is often used as a tool to mitigate clinical impact and viral shedding (Holtkamp et al., 2011). Although specific practices vary across farms, gilts are typically vaccinated before entering the herd, and sometimes the sow herd is mass vaccinated during the year. Most commercial PRRSV vaccines currently sold in the United States are considered “modified live vaccines” (MLV), which means that the vaccine is an attenuated live virus. Vaccines against PRRSV show different degrees of protection against homologous and heterologous challenges (Cano et al., 2007; Díaz et al., 2012; Geldhof et al., 2012); the exact definition of what constitutes a homologous or heterologous challenge is often not clear, especially taking into consideration the genetic diversity existing within PRRSV Type 2 (Shi et al., 2010b). Five major PRRSV vaccines are commercialized in the United States, each developed using a different wild PRRSV isolate (lineages 1, 5, 7, and 8, with the lineage 5 vaccine being the most widely used historically).

Porcine reproductive and respiratory syndrome virus is known to possess a high mutation rate (Hanada et al., 2005; Brar et al., 2014). Genetic mutations for PRRSV are thought to result from RNA polymerase errors (Murtaugh et al., 2010) and from the lack of proofreading (Kappes and Faaberg, 2015). Coupled to that, genetic recombination events can contribute to PRRSV diversity (Forsberg et al., 2002). Thus, the emergence of new variants of PRRSV is expected to occur potentially through both mutation and recombination. Viral variants can quickly emerge in animals (Goldberg et al., 2003) even after inoculation with a single variant (Chang et al., 2002). Thus, the viral population within an animal can be referred to as a viral cloud or swarm (Lauring and Andino, 2010), which suggests that mutation has a considerable impact in virus diversification even on short time scales. In addition, it is assumed that the immune response removes genetic variants of the virus that it recognizes with high specificity, potentially creating selection pressure favoring antigenically divergent PRRSV variants (Murtaugh et al., 2010). Hypervariable portions of the viral genome may be subject to immune selective pressure (Chen et al., 2016); variation in proteins coded by those sites may play a role in evasion of host immune defenses (Ansari et al., 2006; Darwich et al., 2011). PRRSV vaccines are known to diminish the severity of clinical signs once an infection occurs, but not to prevent an infection from occurring (Lyoo, 2015). At the population scale, it can be expected that most animals have some level of immunity because of the high prevalence of natural infection and widespread use of vaccine. This creates the potential for immune-mediated selection to be a driver of PRRSV diversification and evolution (Murtaugh et al., 2010).

The identification of point mutations that are undergoing positive selective pressure is often interpreted as evidence of increased evolutionary fitness (Kryazhimskiy and Plotkin, 2008). One way to identify such sites is to evaluate dN/dS ratios, which measure the rate at which substitutions at non-synonymous sites (dN) occur relative to substitutions in synonymous sites (dS). Substitutions in synonymous sites are thought to be mostly neutral, but a higher occurrence of substitutions in non-synonymous sites can be interpreted as evidence of selective processes that favor changes in the protein sequence (Kosakovsky Pond and Frost, 2005). Positive selective pressure in sites that code for epitopes recognized by the host immune system are of special interest, because they suggest that the origin of such selective pressure, if present, could be driven by the host immune response.

The rapid evolution of PRRSV coupled with the periodic emergence of new and sometimes more virulent viral variants creates a need to continually update our knowledge on circulating PRRSV variants. Reports that show the waxing and waning of different viral types in the whole North America (Shi et al., 2010b) are helpful when understanding continent-wide status of PRRSV lineages. However, understanding viral dynamics on a regional scale could provide important insights into local evolutionary and ecological dynamics of PRRSV, including an improved understanding of how often new variants emerge or re-emerge within the region. Here, we describe the temporal dynamics of PRRSV occurrence in a swine-dense region of the United States, characterizing these patterns according to *ORF*5 genetic lineages and sub-lineages. We quantify the contemporary occurrence of each lineage, investigate the temporal dynamics and turnover of lineages, identify emerging sub-lineages, and examine evolutionary patterns for evidence of positive selective pressures.

## Materials and Methods

Sequences available through the Morrison Swine Health Monitoring Project (MSHMP) were used for this analysis. Briefly, MSHMP is an ongoing voluntary producer-driven nation-wide monitoring program for endemic swine diseases that affect the U.S. swine industry. Based at the University of Minnesota (UMN), this program collects weekly reports on the infection status of sow farms from participating swine-producing companies, veterinary practices, and regional control programs, which serves to capture the occurrence of infectious diseases in the country (Tousignant et al., 2015a, b; Perez et al., 2016). Infection status data classifies farms into the following categories (Holtkamp et al., 2011): Status 1: positive-unstable, Status 2: positive-stable, either through use of live virus inoculation (2lvi) or use of vaccines (2vx); Status 3: provisional negative; and Status 4: negative. The main difference between positive-unstable (Status 1) and positive-stable (Status 2vx or 2lvi) is that unstable herds have an active clinical outbreak and are weaning PRRSV RT-PCR positive piglets. In contrast, PRRSV may be still present in positive-stable herds (through use of field virus inoculation or modified live vaccine) but clinical disease is controlled and piglets weaned from such farms are PRRSV-negative as a result of herd immunity, decreased shedding, and maternal antibodies (Holtkamp et al., 2011). MSHMP collects farm-level data from approximately 3.2 million sows, which represents approximately 50.5% of the United States breeding herd population (National Agricultural Statistics Service [NASS], Agricultural Statistics Board, and United States Deparment of Agriculture [USDA], 2018). Specific production systems (companies involved in pig production) participating in the project also share the *ORF*5 PRRSV sequences identified on their farms as part of routine veterinary management. For example, samples may be submitted by veterinary practitioners to determine if circulating PRRSV on the farm is the same or different from the vaccine virus or a previous variant present on the farm.

For this analysis, we analyzed 4,390 sequences reported between 2009 and 2017 from MSHMP participants located in a relatively isolated swine-dense region in the United States with an approximate area of 250 thousand square kilometers. Production systems operating in this region account for ∼12% of the United States sow population. Approximately 90% of farms within this region participate in MSHMP and in this project in particular. Sequences used in this study came mostly from sow (64.9% of sequences), nursery (16.8%) and finisher farms (14.7%), followed by boar stud farms (0.3%) and sequences without a description of their origin (3.3%). Sequences shared with us by project participants were sequenced according to standardized protocols adopted by laboratories at SDSU (Animal Disease Research and Diagnostic Laboratory et al., 2017), ISU (Zhang et al., 2017) and Eurofins Genomics. Of the *ORF5* gene sequences used in this analysis, seven had fewer than 550 nucleotides. These were deemed incomplete and were excluded from further analysis. We also included 841 *ORF5* gene sequences previously classified into nine different genetic lineages (Shi et al., 2010a, b) and added these to the collection of MSHMP sequences. These sequences, assembled from a database of sequences that spanned from 1989 to 2008, were used as guides to classify the MSHMP sequences into the previously described genetic lineages, and will be referred to here as “anchor” sequences. We also obtained the *ORF5* gene sequences for five vaccines (Ingelvac PRRSV ATP – GenBank ID DQ988080.1, Ingelvac PRRSV MLV – GenBank ID AF066183.4 (both from Boehringer Ingelheim), Fostera PRRSV from Zoetis – GenBank ID KP300938.1, Prime Pac PRRSV RR from Merck – GenBank ID DQ779791.1, and Prevacent, from Elanco – GenBank ID KU131568.1). The Ingelvac PRRSV ATP and Fostera vaccines use isolates belonging to lineage 8, while Ingelvac PRRSV MLV uses a lineage 5 isolate, Prime Pac a lineage 7 isolate and Prevacent a lineage 1 isolate. We also obtained two PRRSV prototypes (Lelystad – GenBank ID NC_043487.1, and VR2332 – GenBank ID EF536003.1, which represent the prototypical European Type 1 and North American Type 2 viruses, respectively). The sequence dataset used here is available in Genbank under the accession numbers MN498289 – MN502669.

**FIGURE 1:**
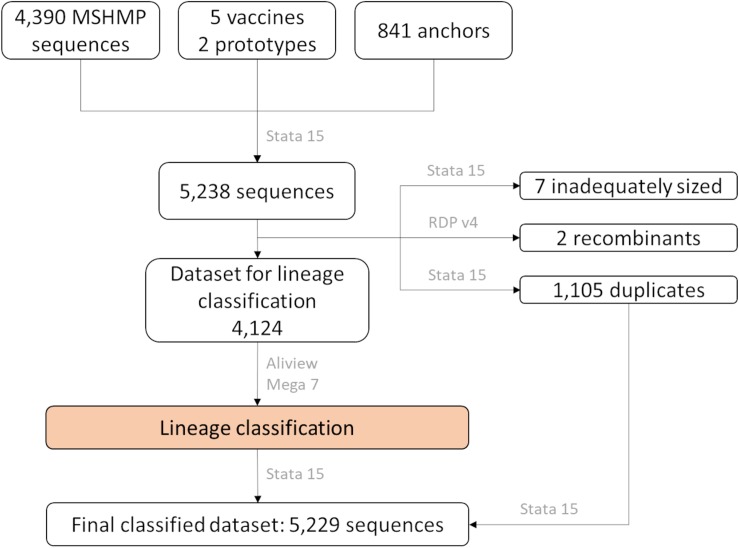
Number of *ORF5* sequences according to their source and how they were treated in the lineage classification process. In gray, name of software used in each step.

Sequences were aligned using the MUSCLE algorithm implemented in AliView (Larsson, 2014) using default settings. The alignment was then examined for the presence of recombinants using the Recombinant Detection Program version 4 (Martin et al., 2015), followed by removal of potential recombinants. In addition, duplicated sequences (with 100% nucleotide similarity) were identified and set aside for the allocation of sequences into lineages. The aligned and cleaned dataset was imported into Mega 7 (Kumar et al., 2016), where the genetic pairwise distance was measured as a percentage nucleotide difference. Using Stata 15 (StataCorp, 2017), each of the MSHMP sequences were assigned to the lineage that had the smallest genetic distance to an anchor. After sequences were classified into lineages, the duplicated sequences were allocated to their respective lineage group according to the sequence with 100% similarity that was kept in the lineage classification process. A flow-chart of these steps can be seen in Figure 1.

A maximum likelihood phylogenetic tree illustrating genetic relatedness of sequences was constructed based on 1,000 bootstraps, adopting the Tamura-Nei model for substitution of amino acids (Tamura and Nei, 1993; Kumar et al., 2016). ClusterPicker software was used to further stratify the most abundant lineage into sub-lineages (Ragonnet-Cronin et al., 2013), in a matter that seemed consistent with the tree main branches while still returning epidemiological meaningful sub-lineages. The phylogenetic tree was then colored according to the lineage classification and source of sequences (anchor versus MSHMP) using Microreact (Argimón et al., 2016). Traditional bootstrap support is estimated based on resampling and replication, which tends to yield low support particularly on deep branches and in large trees with hundreds or thousands of sequences (Lemoine et al., 2018). Branch support on the phylogenetic tree thus was evaluated using the bootstrap support by the transfer method (Lemoine et al., 2018). This method circumvents issues of traditional bootstrapping by assigning a gradual “transfer” index to each clade within the tree rather than a binary presence/absence index for the presence of a clade in each bootstrap (i.e., a clade is considered absent in the bootstrap replicate if the sequences found within the clade is different by even a single member). Temporal changes in the frequency of different lineages was tabulated by quarter of the year. Graphs representing the relative frequency of PRRSV lineages over time were constructed using Stata 15. The frequency with which each lineage occurred over different years was compared using trend analysis for proportions (using the *ptrend* command) in Stata 15 (StataCorp, 2017). For this test only, lineages with fewer than 10 sequences overall were grouped.

The ratio of synonymous to non-synonymous mutations (dN/dS) for all sites in the *ORF5* gene region was calculated using the Single-Likelihood Ancestor Counting protocol (Kosakovsky Pond and Frost, 2005), implemented on the Datamonkey webserver (Pond and Frost, 2005). Because the analysis can only be performed on 500 sequences at a time, the analysis was repeated on ten random subsets of 500 sequences (after removal of 100% identical sequences). Sites were considered under positive selective pressure if the *p*-value associated with a higher rate of non-synonymous versus synonymous mutations was smaller than 0.05. The dN/dS (re-scaled for branch length) of all sites from different runs were averaged and the percentage of runs in which each codon was identified as under significant positive selection was calculated.

## Results

### Lineage Classification

**FIGURE 2:**
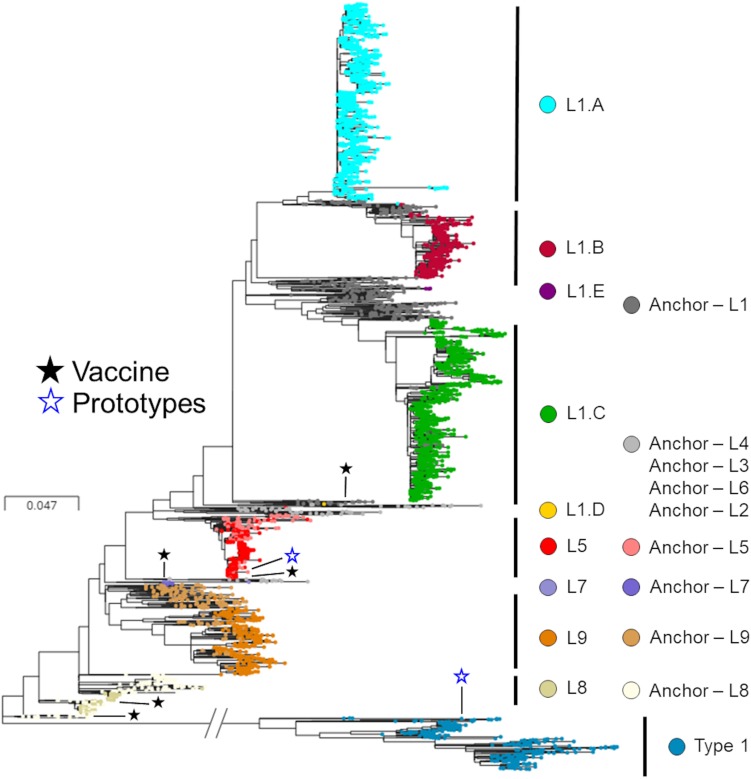
Phylogenetic tree of *ORF5* sequences stratified according to lineages. Colors represent different lineages or sub-lineages, and differences in hues within a color represent anchor versus MSHMP sequences. Prototypes (North American – VR2332; European – Lelystad) and vaccines are highlighted using hollow blue stars and solid dark stars, respectively.

After removal of the seven inadequately sized and two recombinant sequences from the MSHMP data, the remaining 4,381 MSHMP sequences were classified in five different lineages. 70.9% (3,110 sequences) were classified as lineage 1, 10.0% (436) as lineage 5, 0.2% (9) as lineage 7, 2.2% (94) as lineage 8, and 9.2% (404) as lineage 9. A group of 7.5% (328) of the MSHMP sequences were genetically closer to the European Prototype (Lelystad) reference, and were thus classified as Type 1 PRRSV sequences. Lineage 1 was further separated into five sub-lineages (A to E). Out of the total 3,110 sequences in lineage 1, 48.7% (1515) were classified in lineage 1A, 13.9% (433) in lineage 1B, 37.2% (1157) in lineage 1C, 0.03% (1) in lineage 1D and 0.1% (4) in lineage 1E. The phylogenetic tree with all sequences used in the analysis can be seen on Figure 2. Using the Booster method (Lemoine et al., 2018), branch support on main branches (lineages and sub-lineages) was above 90%. The within- and between-lineage nucleotide pairwise genetic distance is shown in Table 1. In general, between lineage/sub-lineage distances are higher than within lineage variation. The distances between sub-lineages of lineage 1 seem to be smaller between them than between other lineages. Broad tree topology was similar when the tree was constructed using nucleotides or amino acids alignment (Supplementary Figure S2).

**TABLE 1:** Mean *ORF5* genetic distance as percentage difference in nucleotides within- (gray cells) and between-lineages (white cells).

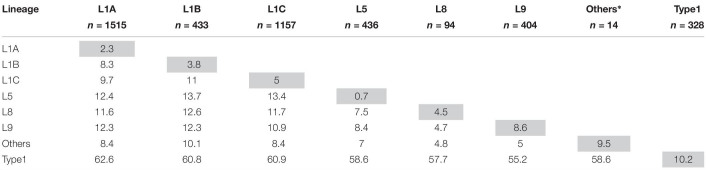

*^∗^Represent the sum of uncommon lineages/sub-lineages, namely sub-lineages 1D (*n* =1), 1E (*n* =4) and lineage 7 (*n* =9).*

### Temporal Dynamics of Lineage Occurrence

**FIGURE 3:**
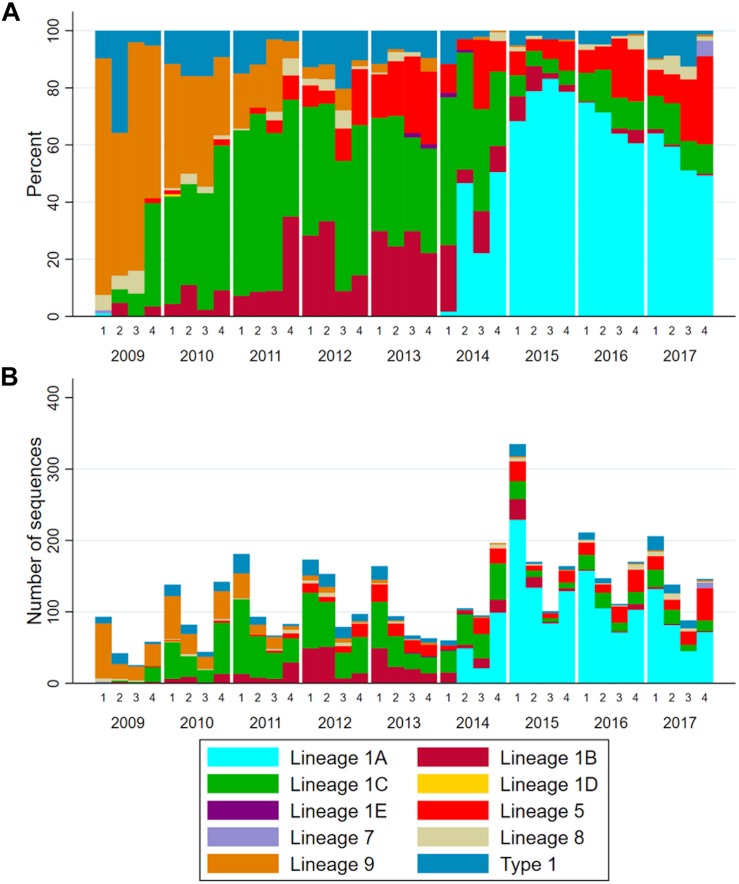
**(A)** Stacked bar chart of the relative frequency and **(B)** number of *ORF5* sequences according to lineages over years and quarters.

On average, the total number of sequences reported to MSHMP increased by 46 each year (Supplementary Table S1), and there was a clear seasonal pattern (Figure 3B). The first quarter of each year (January – March) was the one with highest number of sequences reported in all but 1 year. The relative frequency of each lineage changed through time (Figure 3A and Supplementary Table S1), and specific patterns are noteworthy. First, the absolute and relative occurrence of lineage 9 decreased over time from 68.4% (149 sequences) in 2009 to <1% (5 sequences) in the years 2014–2017. As lineage 9 occurrence declined, lineage 1 occurrence increased until it represented >60% of sequences reported in the period spanning 2011–2017. Within lineage 1, turnover in the dominant sub-lineages is apparent as the relative frequency of lineage 1C between 2009 and 2011 rose from 11.5% to 55.2%, then subsequently declined to approximately 10% of the sequences reported in years 2014–2017. Somewhat concurrently to the emergence of sub-lineage 1C, sub-lineage 1B increased from 1.8% to 27.4% in 2013, then subsequently declined to <2% of sequences reported in 2016 and 2017. Concomitant with the decrease in occurrence of lineage 1C and 1B was a sharp increase in the occurrence of lineage 1A. A single sequence of lineage 1A was observed in 2009, after which this sub-lineage was not detected in any subsequent years until 2014, at which point it was responsible for 37.3% of the sequences. By 2015, almost 75% of sequences belonged to this sub-lineage. Since then, the frequency in which this lineage has occurred decreased (68.4 and 57.3% of the sequences from 2016 and 2017, respectively).

To determine whether changes in sampling effort across time impacted general patterns observed here, we repeated the analysis five times, each time randomly sampling 50 *ORF5* sequences per quarter. General patterns of lineage occurrence did not change, suggesting that patterns of lineage occurrence were not affected by sampling effort in each quarter (Supplementary Figure S1).

The visual patterns and turnover of lineages apparent in Figure 3A were shown to be statistically significant. The increase in the frequency of lineages 1A, 5, 9, and type 1 (*p* < 0.001) was significant, and changes in the grouped frequency of other lineages (a sum of lineages 1D, 1E, and 7, *p* = 0.0472) was also significant, but with a difficult interpretation since this is an aggregate of several uncommon lineages. Lineages 1B and 1C increased in frequency and then decreased (*p* < 0.001). Lineage 9 frequency decreased over time (*p*-value < 0.001), while lineage 8 occurrence remained unchanged (*p*-value = 0.958).

### Evidence for Positive Selective Pressure

**FIGURE 4:**
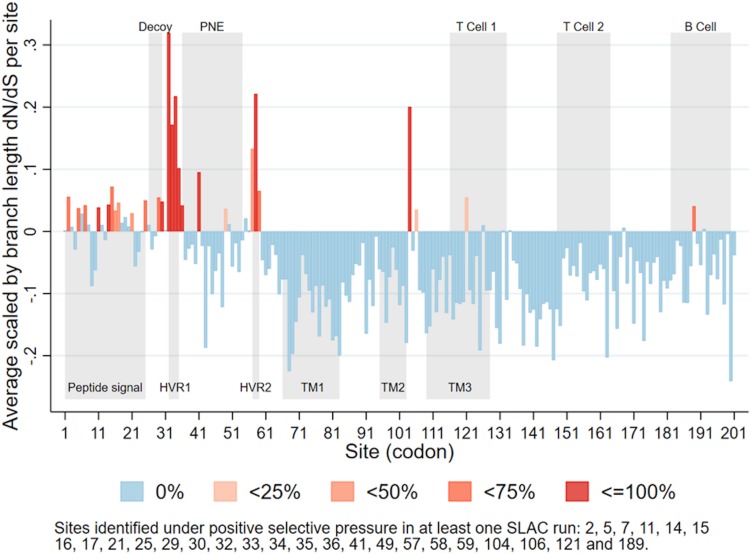
Average scaled by branch length dN/dS for each site in PRRSV ORF5, categorized according to percentage of runs (*n* = 10) in which each site was identified as under positive selective pressure. Upper gray rectangles show antigenic regions (PNE – principal neutralizing epitope), lower gray rectangles show biologically significant regions (HVR – hypervariable region; TM – transmembrane region) (Delisle et al., 2012).

A total of 26 sites were identified as under positive selection in at least one Single-Likelihood Ancestor Counting run (Figure 4). Some sites were identified as under positive selection in all 10 runs, while others were only identified in some runs. Those identified in all runs (with the largest p-value across all runs), were sites 14 (*p*-value = 0.045), 30 (*p*-value = 0.012), 32 (*p*-value < 0.001), 33 (*p*-value < 0.001), 34 (*p*-value < 0.001), 35 (*p*-value < 0.001), 58 (*p*-value = 0.005), and 104 (*p*-value = 0.029). A list of all sites identified as under positive selection in at least one run can be found in the caption of Figure 4. Most of the sites positively selected were located in the first third of the PRRSV ORF5.

**FIGURE 5:**
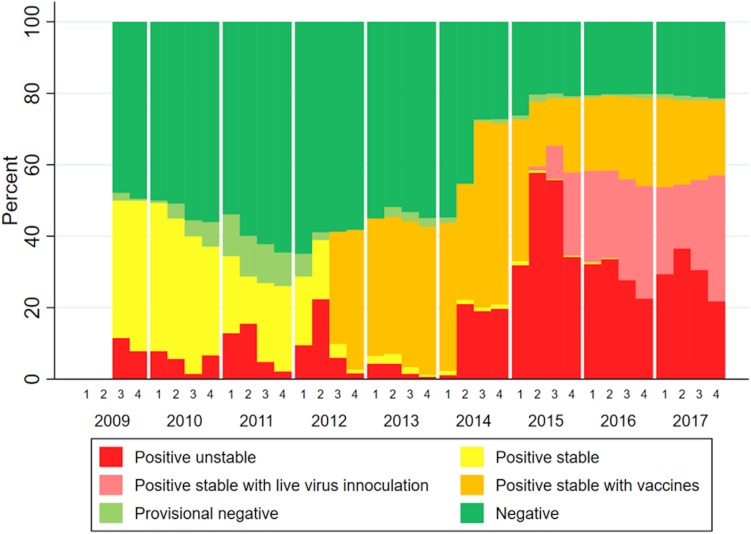
Infection status of farms in the study area over time (quarters and years).

The infection status of farms part of MSHMP in the studied area over the study time span is shown in Figure 5. This data show two periods in which vaccine usage increased, the first one in mid-2012, and a second in approximately mid-2014. Not all farms that reported its status to MSHMP contributed to sequences to this analysis.

## Discussion

We documented the circulation, emergence and sequential turnover of multiple PRRSV lineages in a single United States swine-producing region over a span of 9 years (2009–2017). By classifying over 4,000 PRRSV *ORF5* contemporary sequences into phylogenetic lineages based on pre-2008 data (Shi et al., 2010a, b), we illustrated the continual diversification and temporal dynamics of the PRRSV population. Through further stratifying lineage 1 into three main sub-lineages, we also describe the rapid emergence of a sub-lineage (1A), which was absent in the pre-2008 analysis even though that dataset was based on >8000 sequences from across the world (including the region in which we collected our samples) (Shi et al., 2010b). We also identified sites within PRRSV *ORF5* gene and resultant ORF5 protein that showed evidence of positive selective pressure, indicating that non-synonymous mutations that lead to amino acid changes in the protein at these sites are favored.

From 2009 to 2010, lineage 9 was the most prevalent genetic group observed in our dataset. Shi et al., 2010a, b showed that lineage 9 was rapidly increasing in genetic diversity, which is a proxy for the effective population size of the virus, from 1992 to 2008, and reached a peak from 2004 to 2008. Our data suggests that, at least for our study region, the occurrence of lineage 9 peaked pre-2009, after which it rapidly declined and was replaced mostly by lineage 1 variants. From 2011 to 2017, three different major sub-lineages within lineage 1 emerged, two of those being the most prevalent lineage in certain years (1C from 2011 to 2014, 1A from 2015 to 2017). The emergence of sub-lineage 1A, beginning in 2014 and peaking in 2015 was perceived by veterinarians in the studied area as being a noteworthy event coinciding with the spread of the 1-7-4 RFLP-type. In our dataset, 70.7% of the sequences belonging to the 1A sub-lineage were RFLP-typed as 1-7-4 (followed by 9.4% of sequences with RFLP 1-6-4 and less than 5% of 1-21-4, 1-7-3, 1-4-4, 1-7-2 and several others with less than 1% – see Supplementary Table S2).

While the failure to achieve consistent and reliable PRRSV control and prevention through vaccination demonstrates gaps in our understanding of PRRSV immunology (Murtaugh, 2004), based on current understanding, PRRSV vaccines are expected to better protect against wild viral variants that have a higher degree of similarity to the original parental isolate used for vaccine development (Cano et al., 2007; Díaz et al., 2012; Geldhof et al., 2012). Despite our limited understanding of heterologous cross-protection for PRRSV, the emergence and sequential dominance of different variants leading to lineages and sub-lineages is consistent with the theory of multi-strain dynamics (Gupta et al., 1998; Kucharski et al., 2016). Immune responses, whether originating from human interventions or accumulation of immunity toward wild variants, can exert selective pressure that can ultimately lead to the emergence of new pathogen sub-populations (Gupta et al., 1998). As a virus evolves, immune responses generated against a past variant are expected to become less effective, resulting in a highly complex system, with different lineages interacting through the partial cross-immunity that they generate in the host population (Gupta et al., 1998; Kucharski et al., 2016). Theory predicts that due to frequency-dependent selection amongst co-circulating viral variants, rare antigenic variants are expected to spread more widely in the host population but then subsequently decline as herd immunity rises. Such dynamics have been more thoroughly understood for Influenza A (Webster et al., 1992; McCullers et al., 1999; Ferguson et al., 2003; Nelson et al., 2008) and HIV (McMichael et al., 2010).

For PRRSV, recent research demonstrates that antibodies can exert a strong selective pressure to viral pathogens by targeting specific viral sub-populations, while allowing for the establishment of other sub-populations (Wang, 2016). When comparing PRRSV genetic diversity before and after vaccine adoption in South Korea, PRRSV vaccination was suggested to increase viral genetic heterogeneity and the emergence of new glycosylation sites in viral populations (Kwon et al., 2019). However, the extent in which PRRSV immunity, whether from natural infection or vaccination, can potentially drive the evolution of the virus in the field remains largely unanswered. Our data does show a dominance of non-vaccine related lineages over time, which leads to speculation that these lineages have partially escaped the immunity induced by commercial vaccines or natural infection by variants in other lineages. PRRSV vaccines do not protect against infection (Scortti et al., 2006), but diminish clinical signs and improve animal performance (Cano et al., 2007). Since our project did not evaluate clinical signs of animals, it is difficult to assess the effects of vaccination in that regard. However, despite high region-wide vaccine usage from 2012 onward (Figure 5), Lineage 1A spread widely in the studied region, suggesting that vaccination and other biosecurity measures were insufficient to limit the transmission of lineage 1A.

Lineages shown (Figure 2) and discussed here and elsewhere are based on phylogenetic relationships in the ORF5 region, and might not be predictive of cross-protection and immunological responses developed by hosts when faced with viruses belonging to different lineages. Despite that, the lineage classification protocol used in this study did reveal temporal patterns consistent with what is expected based on epidemiological theory related to the spread of disease in immunologically naive populations. For example, epidemic-shaped curves of occurrence of different PRRSV populations were seen, a pattern consistent with the spread of new pathogens (or subtypes) within a naive population. New (sub-) lineages may potentially be able to become the dominant PRRSV in the population if they are sufficiently immunologically distinct to overcome herd immunity, and herds with different levels of immunity induced by pre-exposure protocols or natural infections might create selective pressure that changes how fast a new viral variant is selected in that population. For PRRSV, it is apparent that protection against homologous PRRSV is more robust than against heterologous variants, though the definition of what constitutes a heterologous virus is highly variable (Cano et al., 2007; Díaz et al., 2012; Geldhof et al., 2012). At the same time, genetic distance has not been shown to correlate with cross-protection, perhaps because pairwise nucleotide identity fails to capture key mutations that impact cross-protection. Studies that further explore the immunological cross-reactivity among PRRSV lineages are needed.

With the data available in this study, it was not possible to investigate the occurrence of specific lineages with vaccination use and more precisely to which vaccine each farm/system used or to which virus was circulating previously on a specific farm. MSHMP data of farms from systems that contributed sequences to this paper (Figure 5) show two periods in which vaccine usage increased. The first increase in mid-2012, and a second in approximately mid-2014. The second spike in vaccine usage coincided with when lineage 1A began spreading in the study area. It is possible that this second spike in vaccine usage was a reaction to the shift in circulating lineages (more specifically, to the emergence of lineage 1A PRRSV). It is also possible that the increased use of vaccines 2012 onward (shown on Figure 5) and the occurrence of lineages 1B and 1C (shown on Figure 3) immunologically selected sequences in a manner that allowed for the emergence of lineage 1A in 2014. By mid-2015, a proportion of farms began using live virus inoculation (lvi). This strategy refers to the use of controlled exposure in gilts through inoculation with live virus isolated from recent clinical outbreak(s) at the farm (Desrosiers and Boutin, 2002). The rationale is that by exposing gilts to virus found in a farm, gilts will mount “homologous” immunity to that specific wild-type virus and contribute to herd immunity and thus stability. According to veterinarians in the area, the increased use of lvi was due to the circulating virus being “different enough” from the viruses used in commercial vaccines. The practice of lvi in the systems here reported began primarily in 2015 (Figure 5). It is difficult to assess the impact that lvi might have on immunologically selecting for specific viral populations within specific lineages, especially with the aggregated data used in this analysis. While the inability of vaccination to control the spread of PRRSV lends credence to immunological selection as a driver of PRRSV diversification (Murtaugh et al., 2010), the impacts that immune-driven selection could have on long term PRRSV evolution remain unknown. Recording exposure procedures (lvi or vaccine use) within farms is crucial when trying to interpret longitudinal patterns of occurrence of PRRSV. In future research aimed at more robustly testing hypotheses about immunity as a driver of evolutionary change, this crucial information would allow for investigation of frequencies in which specific lineages occur in farms pre- and post-vaccine/lvi adoption.

Within ORF5, we found sites under positive selective pressure within or near two hypervariable regions (Figure 4; Hanada et al., 2005; Delisle et al., 2012) located near the principal neutralizing epitope (PNE). The PNE is located between amino acids 36–52 and forms an ectodomain which triggers antibodies development during PRRSV infection (Plagemann et al., 2002; Hanada et al., 2005). The flanking hypervariable regions can be linked to the development of an immune response that block accessibility of antibodies to the PNE (Popescu et al., 2017), including N-linked glycosylation sites such as N34, N44, and N51 (Ansari et al., 2006). In general terms, glycosylation may modulate protein-protein interactions, whether these proteins involve the humoral or cellular immune response of the host (Lisowska, 2002). In PRRSV, there is evidence that these glycosylation sites play a role in glycan shielding, which is an important mechanism by which the virus evades neutralizing immune responses (Vu et al., 2011). While our findings do not explicitly explain the change in lineage, it does raise one hypothesis of the mechanism behind such change. Further studies on how specific portions for the genome, both within ORF5 and the whole genome, modulate immune recognition and possibly selective pressure are needed.

**FIGURE 6:**
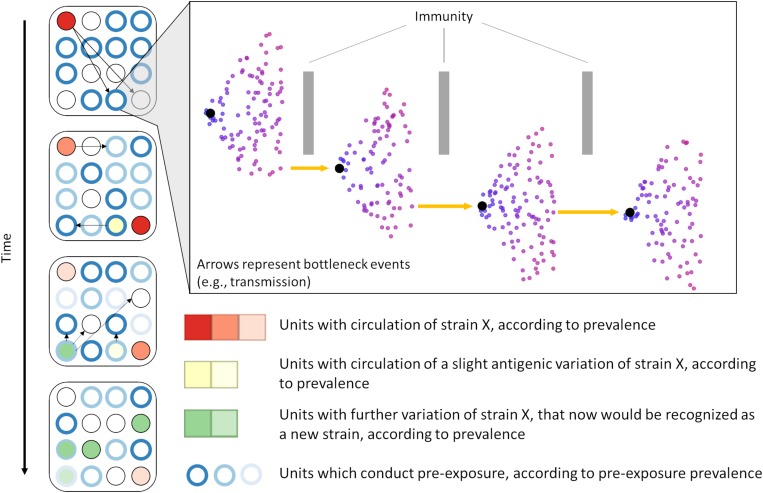
We hypothesize that PRRSV evolution is partially driven by immune-mediated selective pressure. Immune-mediated pressure (either within an animal or during transmission between animals/farms) selects for escapee viral variants (inset). Over time, the selection of escapees may allow for emergence of a heterologous viral populations (i.e., strains, genetic groups, or lineages) which are able to spread within the host population. In scenarios in which some method of pre-exposure is adopted, prevalence of immunity against specific types of PRRSV is high (often artificially through vaccination or live virus inoculation) despite high population turnover, possibly favoring the occurrence of immune-mediated selection.

We also consistently identified positive selective pressure within the PNE region, specifically for amino acid 41. The identification of positive selective pressure in this region suggests that viral variants with different amino acid composition in that region may experience higher fitness and thus are favored. Since this region seems to be the primary binding site of neutralizing antibodies developed during PRRSV infection (Plagemann et al., 2002; Kim et al., 2013), this suggests that the reason for such selective pressure could be immune in nature. Such a scenario has been considered as a possible explanation for long-term evolution of RNA viruses (Domingo et al., 1996; Pérez-Sautu et al., 2011). Additional *in vitro* research is necessary to further clarify the immunological importance of sites identified in our analysis. However, our results suggest the plausibility of a scenario where PRRSV variants with mutations in key immunological regions are able to evade immune responses and thus persist and spread within host populations with partial immunity (Figure 6). Further studies to investigate the role of an incomplete immunity on the evolution of PRRSV are required.

Other mechanisms that might change the ability of the virus to infect hosts have also been proposed. Non-muscle myosin heavy chain 9 (MYH9) is a molecule that has been shown to be an essential host factor for PRRSV infection (Gao et al., 2016). MYH9 interacts with PRRSV glycoprotein 5 (coded for by ORF5), changing cell susceptibility to infection. Further studies that investigate the contribution that molecules such as MYH9 have on the infection of different ORF5 PRRSV variants are needed. Additionally, non-neutralizing antibodies can delay the induction of neutralizing antibodies (Ostrowski et al., 2002) in PRRSV infection. Indeed, the mean level and duration of viremia in pigs was greater among animal injected with sub-neutralizing PRRSV-specific IgG antibodies (Yoon et al., 1996), suggesting the existence of an antibody-dependent enhancement (ADE) effect in PRRSV. The extent in which prior exposures to the virus can elicit such effect, and how this may relate to emergence of new viral variants, also remains uncertain.

As an epidemiologic study relying on secondary data generated at the population level, this study has several limitations. Our sequence data were generated by different production systems that differ in number of farms, number of samples submitted, management practices, and health monitoring protocols. Because of that, information may be incomplete and interpretation of data might not always be straightforward. For example, the reason for sample collection (clinical outbreak or routine monitoring), sample composition (single versus pool of animals) and type of sample (serum or tissues) is not always clear. The lack of a denominator (total amount of animals sampled in a farm, total number of farms tested) does not allow for the calculation of risk indicators for disease occurrence. Data contribution by each system also varies with time. However, restricting the data to only the periods in which all systems contributed to the dataset would limit our ability to visualize long-term trends. Additionally, the production system that was responsible for 79% of all sequences was present in the study for the entire study period. Therefore, we believe that biases introduced by this issue were likely small and would not have changed the conclusions of our work. In this United States region, systems that participate in the MSHMP represent approximately 90% of the swine farms. The remaining 10% of farms belong to smaller systems in the area or independent farmers. By having data from systems that represent the vast majority of farms in this region, we expect our data to be reasonably representative of PRRSV occurrence in the region as a whole. Additionally, despite the shortcomings mentioned above, the usage of MSHMP data allows us to work with data directly from the systems, which might suffer less bias toward diseased animals than usual veterinary diagnostics laboratories data do.

Another limitation of this analysis involves the data generation process for the sequences analyzed here. Production systems usually collect samples and send them to different diagnostic laboratories. Laboratory details on quality of sequence reads were not available. These sequences most likely represent a consensus of viral sub-populations present within the host (Goldberg et al., 2003; Lauring and Andino, 2010), but further information that could help in assessing the quality of the read and the variability of sub-populations is not available. The sequences used here are from the *ORF5* gene alone and may not fully represent evolutionary dynamics elsewhere in the genome, since the *ORF5* gene represents approximately 4% of the whole genome of PRRSV. Studies that further explore whole genome sequencing as a tool to understand PRRSV epidemiological and evolutionary patterns are required.

Factors affecting PRRSV dynamics in specific farms are not clearly understood. We show overall temporal dynamics of PRRSV in a swine-producing region of the United States, however, we have limited farm-level information. Thus, we have limited ability to track turnover of viral variants within farms, though we expect this to be influenced by management practices, such as the vaccination protocol adopted by farms, the movement of animals and personnel to and between farms, the proximity to other swine producing farms, how neighboring farms manage their animals, etc. Pig production in the U.S. swine industry is characterized by multi-site pig production, which refers to segregating the breeding herd from the growing herd such that animals in each stage of production are housed at separate locations. Multi-site production results in the movement of animals between different production sites, which can be located in different states within the United States (Valdes-Donoso et al., 2017; VanderWaal et al., 2018; Kinsley et al., 2019). The role of animal movement in shaping the temporal dynamics of PRRSV lineages is outside the scope of this study, but is an area of active research. In addition, the commingling of animals from different sources, which might have been previously exposed to different viral populations, may allow for the introduction of viral types prevalent in other parts of the country and also exacerbate the potential for recombination of viral populations. Still, in our dataset we found evidence for recombination in only two MSHMP sequences.

### Future Research

Immune interaction between infections of differing PRRSV isolates remains poorly understood in swine. The vast adoption of control protocols that rely on imperfect immune response aimed mostly at reducing severity of upcoming infections (such as pre-exposure protocols with commercial vaccines or with lvi) suggests that a better understanding of the cross-immunity generated by infection with different isolates of the virus would be valuable to the industry as a whole. Prospective studies that obtain sera from sow farms under different pre-exposure regimens and follow the farms through time recording PRRSV occurrence would provide valuable information of potential cross-immunity in field conditions. Of interest also is the better understanding of how the spread different lineages/sub-lineages are related to epidemiological data, for example, animal movement data and farm proximity. This might allow for a better comprehension of drivers for PRRSV transmission while allowing for the evaluation of the effectiveness of practices aimed at reducing PRRSV risk (dead animals disposal, manure composting, filtering the air of farms, to name a few).

This study reflects data from a single United States region, which possibly does not reflect PRRSV diversity and temporal dynamics of the whole swine industry in the country (Shi et al., 2010b). That being said, the data presented here reflects a substantial portion of the U.S. swine industry in a region that is relatively spatially discontinuous from other swine producing regions in the United States. In addition, the general pattern of emergence and turnover of different lineages over time observed here describe an evolutionary phenomenon that is expected to also occur in other United States regions. A better understanding of the natural history of PRRSV can provide insights that can potentially aid in mitigating the impact of the emergence of new viral variants as well as serving as a basis for further work exploring the evolution of PRRSV and the effect this has on disease control, management and impact on the industry.

## Conclusion

Here, we describe the occurrence of PRRSV over 9 years in a single United States region. We identified the emergence and turnover of different lineages and sub-lineages in the commercial pig population. Such rapid turnover in the dominant lineage through time suggests that temporal patterns of PRRSV occurrence are characterized by multi-strain dynamics, where different PRRSV variants potentially interact through immune-mediated competition or selection. However, cross-immunity between different PRRSV lineages elicited by natural or intentional infection is not fully understood, which hinders the effectiveness of disease control. More research is needed on drivers of evolution and emergence of new sub-lineages in order for the industry to be able to predict, prevent, and mitigate the impacts of PRRSV. Ongoing surveillance for PRRSV using molecular epidemiological methods is invaluable to characterize the evolution of the virus but also to identify recent and historical trends that help understanding the natural history of PRRSV in the United States.

## Data Availability Statement

The sequence dataset used here is available in GenBank under the accessions numbers MN498289–MN502669.

## Author Contributions

IP and KV analyzed, conceptualized, and designed the study. CC, JS, CV, and KV contributed to acquisition of the data. IP, CC, AR, JS, CV, DS, and KV interpreted the data. MM aided in early interpretation of data. All authors but MM were involved in drafting the manuscript and revising it critically for intellectual content and have given final approval of the version to be published.

## Conflict of Interest

The authors declare that the research was conducted in the absence of any commercial or financial relationships that could be construed as a potential conflict of interest.
