# Supplementary material for: Temporal Dynamics of Co-circulating Lineages of Porcine Reproductive and Respiratory Syndrome Virus
Source: Front Microbiol. 2019 Nov 1;10:2486. doi: 10.3389/fmicb.2019.02486 (PMC6839445; doi:10.3389/fmicb.2019.02486)
Supplement: Supplementary file 1 [file Table_1.docx]

**Supplementary Table 1.** Absolute and relative frequency (%) of sequences stratified by lineage or sublineage, according to the year sequences were identified.

| **Lineage** | **Number of sequences (%)** | | | | | | | | |  |
| --- | --- | --- | --- | --- | --- | --- | --- | --- | --- | --- |
|  | 2009 | 2010 | 2011 | 2012 | 2013 | 2014 | 2015 | 2016 | 2017 | All years |
| L1.A | 1 (0.5) | 0 (0) | 0 (0) | 0 (0) | 0 (0) | 170 (37.3) | 576 (74.8) | 437 (68.4) | 331 (57.3) | 1515 (34.6) |
| L1.B | 4 (1.8) | 29 (7.1) | 56 (13.2) | 121 (24.1) | 106 (27.3) | 51 (11.2) | 50 (6.5) | 11 (1.7) | 5 (0.9) | 433 (9.9) |
| L1.C | 25 (11.5) | 171 (42.1) | 234 (55.2) | 228 (45.4) | 153 (39.4) | 159 (34.9) | 47 (6.1) | 72 (11.3) | 68 (11.8) | 1157 (26.4) |
| L1.D | 0 (0) | 1 (0.3) | 0 (0) | 0 (0) | 0 (0) | 0 (0) | 0 (0) | 0 (0) | 0 (0) | 0 (0.0) |
| L1.E | 0 (0) | 0 (0) | 0 (0) | 0 (0) | 2 (0.5) | 2 (0.4) | 0 (0) | 0 (0) | 0 (0) | 4 (0.1) |
| L5 | 1 (0.5) | 5 (1.2) | 12 (2.8) | 48 (9.6) | 77 (19.9) | 54 (11.8) | 59 (7.7) | 83 (13) | 97 (16.8) | 436 (10.0) |
| L7 | 1 (0.5) | 0 (0) | 0 (0) | 0 (0) | 0 (0) | 0 (0) | 0 (0) | 0 (0) | 8 (1.4) | 9 (0.2) |
| L8 | 9 (4.1) | 7 (1.7) | 8 (1.9) | 16 (3.2) | 5 (1.3) | 6 (1.3) | 7 (0.9) | 14 (2.2) | 22 (3.8) | 94 (2.1) |
| L9 | 149 (68.4) | 144 (35.5) | 71 (16.8) | 23 (4.6) | 9 (2.3) | 2 (0.4) | 3 (0.4) | 1 (0.2) | 2 (0.4) | 404 (9.2) |
| Type 1 | 28 (12.8) | 49 (12.1) | 43 (10.1) | 66 (13.2) | 36 (9.3) | 12 (2.6) | 28 (3.7) | 21 (3.3) | 45 (7.8) | 328 (7.5) |
| Total | 218 | 406 | 424 | 502 | 388 | 456 | 770 | 639 | 578 | 4381 |
