# Supplementary material for: Temporal Dynamics of Co-circulating Lineages of Porcine Reproductive and Respiratory Syndrome Virus
Source: Front Microbiol. 2019 Nov 1;10:2486. doi: 10.3389/fmicb.2019.02486 (PMC6839445; doi:10.3389/fmicb.2019.02486)
Supplement: Supplementary file 2 [file Table_2.docx]

**Supplementary Table 2.** Absolute frequency sequences according to their RFLP type and lineage.

| RFLP pattern | Lineages | | | | | | | |  |
| --- | --- | --- | --- | --- | --- | --- | --- | --- | --- |
|  | L1.A | L1.B | L1.C | L5 | L8 | L9 | Other lineages* | Type 1 | |
| 1-7-4 | 1,007 | 0 | 5 | 0 | 0 | 1 | 0 | 0 | |
| 1-4-4 | 58 | 0 | 899 | 0 | 1 | 4 | 7 | 0 | |
| 2-5-2 | 0 | 0 | 0 | 362 | 0 | 0 | 0 | 0 | |
| 1-18-2 | 0 | 241 | 0 | 0 | 0 | 0 | 0 | 0 | |
| 1-1-1 | 0 | 0 | 0 | 0 | 0 | 0 | 0 | 240 | |
| 1-4-2 | 0 | 0 | 17 | 0 | 44 | 120 | 0 | 0 | |
| 1-2-2 | 0 | 0 | 0 | 0 | 1 | 141 | 0 | 0 | |
| 1-6-4 | 133 | 0 | 0 | 1 | 0 | 0 | 0 | 0 | |
| 1-26-2 | 0 | 113 | 0 | 0 | 0 | 0 | 0 | 0 | |
| 1-3-4 | 1 | 1 | 85 | 0 | 0 | 1 | 0 | 0 | |
| 1-2-4 | 8 | 1 | 16 | 0 | 0 | 60 | 0 | 0 | |
| 1-3-2 | 0 | 0 | 2 | 0 | 38 | 33 | 1 | 0 | |
| 1-158-1 | 0 | 0 | 0 | 0 | 0 | 0 | 0 | 72 | |
| 1-21-4 | 65 | 0 | 0 | 0 | 0 | 0 | 0 | 0 | |
| 1-7-3 | 60 | 0 | 0 | 0 | 0 | 0 | 0 | 0 | |
| 1-7-2 | 41 | 0 | 0 | 0 | 0 | 2 | 0 | 0 | |
| 1-4-3 | 10 | 0 | 15 | 0 | 0 | 0 | 0 | 0 | |
| 1-13-2 | 0 | 14 | 0 | 0 | 0 | 5 | 0 | 0 | |
| 1-1-2 | 0 | 1 | 0 | 0 | 3 | 14 | 0 | 0 | |
| Ambiguities | 13 | 0 | 3 | 12 | 3 | 0 | 2 | 1 | |
| Others** | 119 | 62 | 115 | 61 | 4 | 23 | 4 | 15 | |
| * Represent the sum of uncommon lineages/sub-lineages, namely sub-lineages 1D (n=1), 1E (n=4) and lineage 7 (n=9) | | | | | | | | |  |
| ** Represents the sum of all other RFLP types and patterns not assigned to a known RFLP type | | | | | | | | |  |
