# Supplementary material for: Temporal Dynamics of Co-circulating Lineages of Porcine Reproductive and Respiratory Syndrome Virus
Source: Front Microbiol. 2019 Nov 1;10:2486. doi: 10.3389/fmicb.2019.02486 (PMC6839445; doi:10.3389/fmicb.2019.02486)
Supplement: Supplementary file 3 [file Data_Sheet_1.docx]

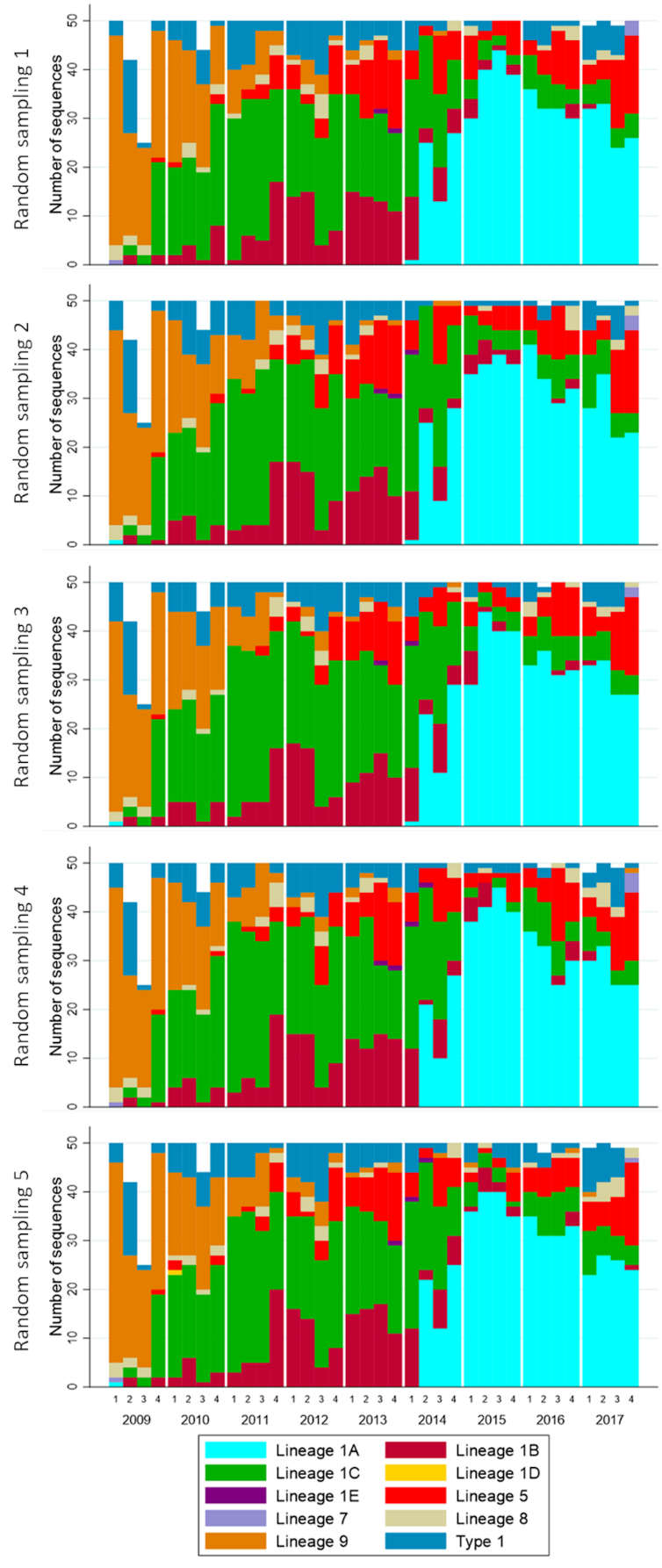


**Supplementary Figure 1**. Random sampling of 50 sequences per quarter. General patterns of lineage occurrence did not change when sampling per quarter was forced to 50 sequences, suggesting that the patterns of lineage occurrence were not affected by sampling effort on each quarter.
