# Supplementary material for: Temporal Dynamics of Co-circulating Lineages of Porcine Reproductive and Respiratory Syndrome Virus
Source: Front Microbiol. 2019 Nov 1;10:2486. doi: 10.3389/fmicb.2019.02486 (PMC6839445; doi:10.3389/fmicb.2019.02486)
Supplement: Supplementary file 4 [file Data_Sheet_2.docx]

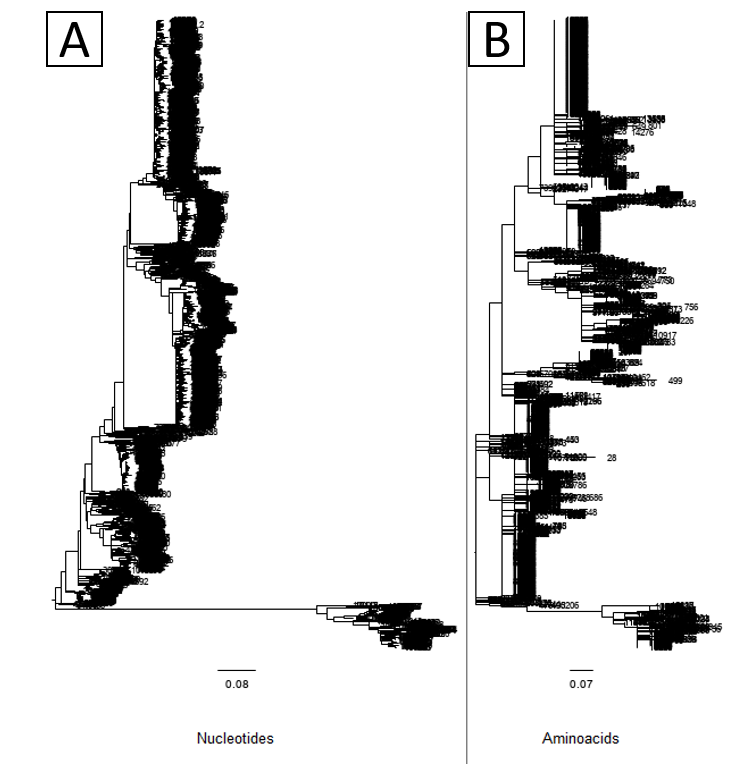


**Supplementary Figure 2**. Tree topology of *orf*5 sequences according to how it was constructed. **A** – tree constructed using a nucleotide alignment and **B** – tree constructed using an amino acid alignment.
